# Glacial Inception in Marine Isotope Stage 19: An Orbital Analog for a Natural Holocene Climate

**DOI:** 10.1038/s41598-018-28419-5

**Published:** 2018-07-05

**Authors:** Stephen J. Vavrus, Feng He, John E. Kutzbach, William F. Ruddiman, Polychronis C. Tzedakis

**Affiliations:** 1 Nelson Institute Center for Climatic Research, University of Wisconsin-Madison, 1225 W. Dayton Street, Madison, WI 53706 USA; 2 Department of Environmental Sciences, University of Virginia, 291 McCormick Road, Charlottesville, VA 22904 USA; 3 Environmental Change Research Centre, Department of Geography, University College London, London, WC1E 6BT England; 4 College of Earth, Ocean and Atmospheric Sciences, Oregon State University, Corvallis, OR 97331 USA

## Abstract

The Marine Isotope Stage 19c (MIS19c) interglaciation is regarded as the best orbital analog to the Holocene. The close of MIS19c (~777,000 years ago) thus serves as a proxy for a contemporary climate system unaffected by humans. Our global climate model simulation driven by orbital parameters and observed greenhouse gas concentrations at the end of MIS19c is 1.3 K colder than the reference pre-industrial climate of the late Holocene (year 1850). Much stronger cooling occurs in the Arctic, where sea ice and year-round snow cover expand considerably. Inferred regions of glaciation develop across northeastern Siberia, northwestern North America, and the Canadian Archipelago. These locations are consistent with evidence from past glacial inceptions and are favored by atmospheric circulation changes that reduce ablation of snow cover and increase accumulation of snowfall. Particularly large buildups of snow depth coincide with presumed glacial nucleation sites, including Baffin Island and the northeast Canadian Archipelago. These findings suggest that present-day climate would be susceptible to glacial inception if greenhouse gas concentrations were as low as they were at the end of MIS 19c.

## Introduction

**Figure 1:**
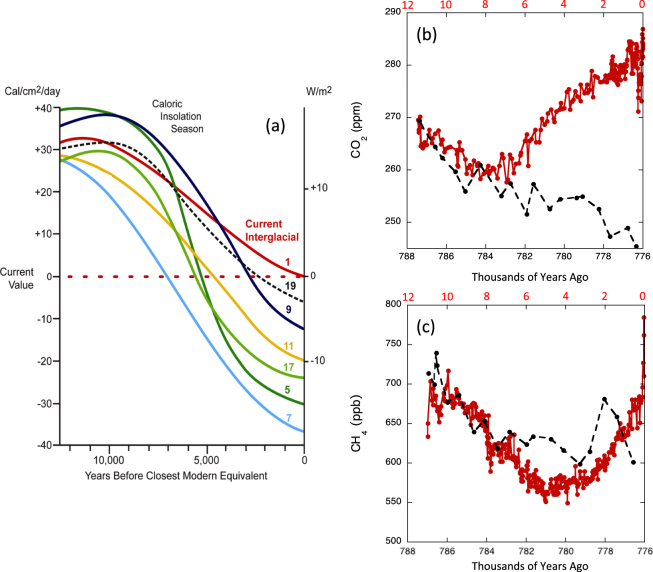
(**a**) Caloric Northern Hemisphere half-year summer insolation for MIS 1, 5, 7, 9, 11, 17, and 19, relative to each stage’s insolation minimum at year 0 [Updated from a previous version^61,62^]. Trends in (b) CO_2_ and (**c**) CH_4_ concentrations^7^ during MIS19 (black) and MIS1 (red). CO_2_ data for MIS19 is from Dome C^6^ and from Law Dome^9,10^ and Dome C^11,12^ for MIS1.

We present a global climate model simulation, using the Community Climate System Model version 4 (CCSM4, see Methods), run on the interglaciation during Marine Isotope Stage 19c (MIS19)^1^ to provide a ground-truth analog for a contemporary climate system not affected by human activities. Both MIS19 and the MIS11 interglaciation have been considered as possible analogs to the Holocene (MIS1) (see Supplementary Information), but MIS19 is regarded as the best analog during the past 800,000 years for which ice-core records are available^2,3^. The insolation histories of MIS19 and MIS1 are very similar, such that their caloric half-year summer insolation differed by no more than 3 W m^−2^ throughout (Fig. 1a). The only significant difference in their orbital parameters is the weaker amplitude of the obliquity signal during MIS19. By the time equivalent to present day, however, the MIS19 obliquity was nearly identical to the modern value, and the precession and eccentricity parameters were also very similar (Table 1). The strong orbital resemblance between MIS19 and MIS1 enables direct comparisons between climatic signals from these two interglaciations, including GHG trends, such that the closest insolation analog to the present day occurred at the corresponding insolation minimum 777,000 years ago at the close of MIS19^3^.

**Table 1:** Orbital parameters and greenhouse gas concentrations^6,7,9–12^ used for the four model experiments in this study.

Forcing	MIS19 (777 ka)	PI (Year 1850)	PI_NAT (Year 1850)	20^th^ Century (1850–2005)
Obliquity	23.3°	23.4°	23.4°	23.4°
Precession	108.9°	102.7°	102.7°	102.7°
Eccentricity	0.023	0.017	0.017	0.017
CO_2_ (ppm)	245	285	245	353
CH_4_ (ppb)	631	792	445	1720
N_2_O (ppb)	270	276	270	310
CFC-11 (ppt)	0	0	0	280
CFC-12 (ppt)	0	0	0	484
Radiative Forcing (W m^−2^)	−0.92	—	−1.04	+1.92

The values for the 20^th^ Century simulation refer to conditions in year 1990. Radiative forcing is relative to the PI simulation referenced to year 1850.

Substantial glacial inception likely began by 777,500 to 775,500 years ago, based on various criteria: the appearance of millennial-scale oxygen-isotopic oscillations in marine cores, the appearance of ice-rafted debris in marine cores, and an assumed delay between these first-appearance criteria and the accumulation of enough terrestrial ice to have driven them^3^. A similar MIS19 end date of 778.4–776.4 ka +/− 2.3 kyr, based on a detailed ^40^Ar/^39^Ar chronology, was estimated from a high-resolution record from Italy^4^.

The comparable orbital configurations but contrasting glaciation histories of MIS19 and MIS1 motivated a recent modeling study^5^ using a low-resolution Earth system model of intermediate complexity (EMIC) to compare the potential for glacial inception in both stages. The model simulated a buildup of large-scale ice volume at the close of MIS19, whereas glaciation during MIS1 depended critically on whether Holocene CO_2_ concentrations followed an inferred natural trajectory to 240 ppm (slightly lower than our estimate) or the actual trajectory of 280–285 ppm by the dawn of the Industrial Revolution.

This paper also investigates the onset of glaciation in MIS19 but does so using a much higher-resolution global climate model (GCM) simulation that represents circulation patterns, energy transport, and hydrologic processes much more realistically than an EMIC. Furthermore, we ran a single, unconstrained simulation, whereas the previous study^5^ first screened a 20-member perturbed-physics model ensemble and selected only the 20% of simulations that matched observations of glacial inception at the end of MIS19 and MIS5 but not MIS1. Our CCSM4 GCM experiment of MIS19 informs whether contemporary climate conditions are suitable for glacial inception under the much lower GHG concentrations estimated in the absence of both ancient and recent carbon emissions from agriculture and industrialization.

Reconstructions of CO_2_ and CH_4_ concentrations from ice core records^6–12^ at Antarctic Dome C, using the EDC3 chronology^13^, can be compared between MIS19 and MIS1. The MIS19 CO_2_ trend reached an early peak of 269 ppm, essentially identical to the peak of 270 ppm early in MIS1, after which both trends started falling (Fig. 1b). The MIS19 trend continued declining through the time of the modern-day equivalent (777,000 years ago), but the MIS1 trend reversed direction 7000–8000 years ago and rose for the remainder of the Holocene. These opposing trends are consistent with the prediction from the Early Anthropogenic Hypothesis (EAH) that MIS1 values would have continued falling if not for early agriculture, as they did in other recent interglacials^14^. The 245-ppm CO_2_ concentration estimated as the modern-day equivalent in MIS19 (see Methods) is very close to the interpolated 248 ppm value from the latest Dome C data^6^ and lies at the top of our previously estimated 240–245 ppm range^14^. The difference in CO_2_ radiative forcing between 245 ppm and 248 ppm amounts to only 0.06 W m^−2^ and thus should have a minor effect on the model simulation.

CH_4_ in MIS19 reached an early-interglacial peak of 739 ppb, compared with the 717-ppb peak early in MIS1, after which both trends began falling (Fig. 1c). The MIS19 concentration then dropped to a value of 631 ppb by the time of the modern-day equivalent at 777 ka, interrupted by a brief CH_4_ maximum near 778 ka that is interpreted as a millennial-scale oscillation because it was defined by two data points separated by just a little over 1000 years^14^. The CH_4_ trend then resumed its gradual orbital-scale downward trend through (and beyond) the time of the modern-day equivalent, dropping to 530 ppb by around 772 ka (not shown). In contrast, the MIS1 CH_4_ trend reversed direction 5000 years ago and has moved steadily upward since then, reaching around 790 ppb just before the industrial greenhouse-gas era began in 1850. These opposing directions (down for MIS19, up for MIS1) are again consistent with the EAH prediction. In this case, however, the MIS19 CH_4_ trend remained higher than the downward path of the MIS1 trend proposed in the EAH, based on the average trend of many recent interglaciations^15^ and estimated Holocene emissions arising from plant and animal domestication (see Methods), but the effect of this difference on radiative forcing is small compared with the influence of CO_2_.

Ideally, the contemporary “natural” CO_2_ concentration in the absence of all anthropogenic carbon emissions would be derived from an interactive carbon cycle model with an accompanying dynamical vegetation model. Unfortunately, neither of these modules is up to the task. Coupled carbon cycle models in GCMs are unreliable in their simulations of the observed industrial-era rise in CO_2_, producing a 45 ppm spread in near-present-day concentration^16^, which is about twice as large as the pre-industrial CO_2_ increase from the mid-Holocene that we are studying. Interactive global vegetation models in GCMs often produce unrealistic distributions of grasses and trees in the present-day climate, highly divergent future vegetation distributions even for the same climatic forcing scenario, and a large spread in their carbon-climate feedback parameters^17–20^. These modeling inadequacies necessitate the use of prescribed estimates of GHG concentrations.

## Results

**Figure 2:**
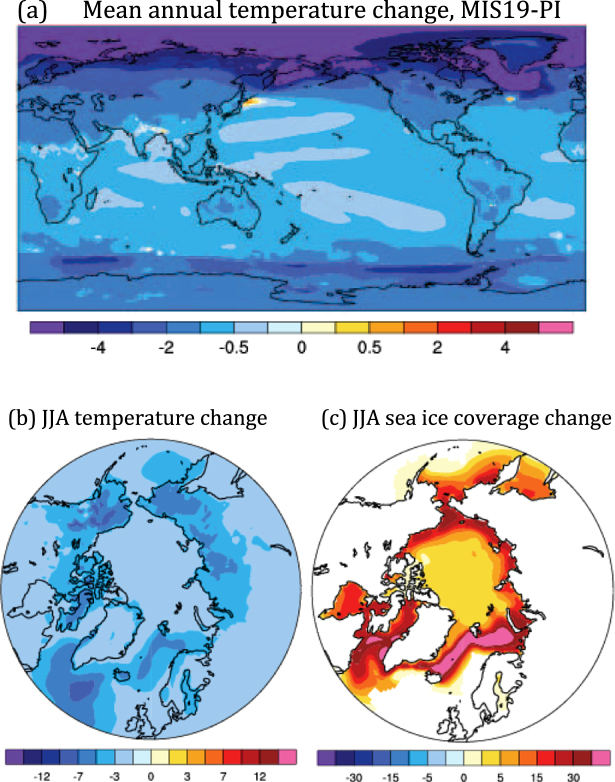
Changes in surface temperature (K) and sea ice coverage (%) between MIS19 and PI. (**a**) Mean annual temperature, (**b**) Boreal summer temperature, June-August, (**c**) Boreal summer sea ice.

We employed the CCSM4 GCM (see Methods) to test whether glacial inception is simulated for the Stage 19 equivalent of the present day at 777 ka (Table 1), noting that the two largest forcings–insolation and CO_2_–are very close analogs to those proposed in the EAH. The negative greenhouse forcing in MIS19, relative to pre-industrial “PI” conditions at year 1850, causes a much colder climate. The mean-annual global temperature falls by 1.27 K, while the 5–6 K cooling in the high Arctic is the most pronounced anywhere (Fig. 2a). Polar temperatures drop the most over sea ice and along adjacent Arctic land, including Alaska and Baffin Island. High-latitude cooling during summer is especially important for promoting glaciations^21^, and the MIS19 simulation produces substantial and widespread summertime temperature decreases of over 3 K across much of the Arctic, especially over northeastern Siberia, northwestern North America, and the Canadian archipelago (Fig. 2b). Accompanying this terrestrial cooling is a comparable temperature decrease over the North Atlantic and Nordic Seas, where a pronounced expansion of sea ice occurs (Fig. 2c). The cooling in the North Atlantic versus the North Pacific is presumably enhanced by the response of the Atlantic Meridional Overturning Circulation (AMOC), which weakens by 2 Sv (25.5 Sv to 23.5 Sv) in MIS19.

**Figure 3:**
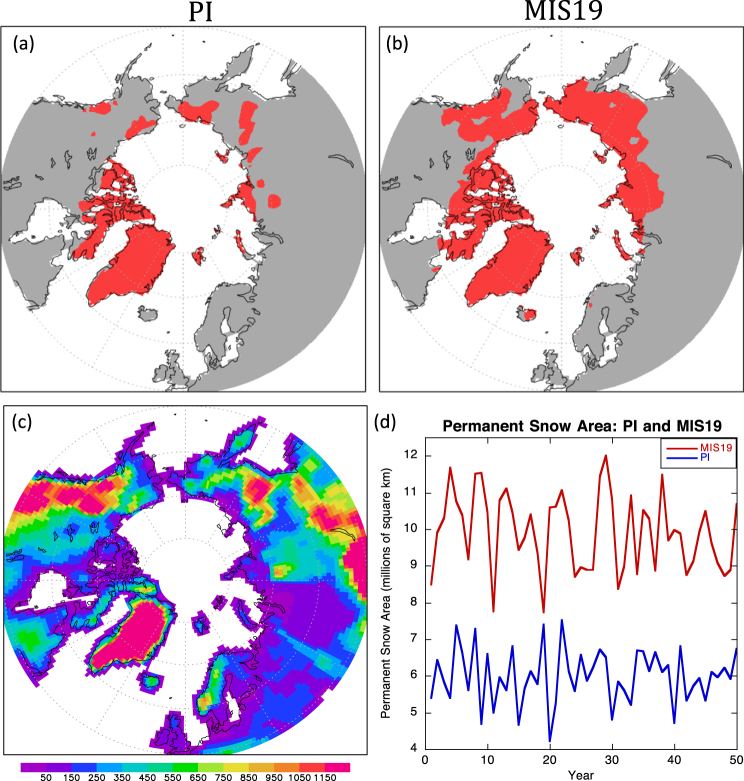
Regions with a year-round snow pack in (**a**) PI and (**b**) MIS19, based on August snow cover of at least 5% in a gridbox. (**c**) Model topography, meters. (**d**) Area of year-round snow cover in the Northern Hemisphere in final years of PI and MIS19 simulations.

These regional summertime cooling maxima of over 5 K associated with expansions of terrestrial snow cover and marine ice cover are both expressions and drivers of incipient glaciation. One way to identify year-round snow cover is to apply the 5% grid-box snow concentration threshold (see Methods) to the PI and MIS19 simulations (Fig. 3). Based on this definition, much more permanent snow cover emerges in MIS19, especially in Siberia, Alaska, and the Canadian Northwest Territories. Overall, snow cover persisting throughout the year encompasses 9.92 million km^2^ in MIS19, compared with 5.99 million km^2^ in PI, representing a 66% increase (92% expansion excluding Greenland). Topography exerts a strong influence on where permanent snow cover forms (Fig. 3c), particularly in the PI simulation, as highly elevated regions are the preferred sites in Siberia and Alaska (Brooks Range and coastal Alaska Range). Elevation plays a less obvious role in the MIS19 run, which is cold enough to support a year-round snow pack even at lower altitudes. We assume that these regions of year-round snow cover would eventually become land ice if the model included glacial processes.

Based on our standard definition of glacial inception (see Methods), the MIS19 simulation reaches this state, because every year at the end of the MIS19 run has more extensive year-round snow cover than PI (Fig. 3d). Conversely, the PI simulation is not cold enough to constitute a state of glacial inception relative to CCSM4’s transient 20^th^-century simulation (Table 1), because several years in the 20^th^-century run exhibit more permanent snow cover than the year with the least permanent snow cover in PI (not shown).

**Figure 4:**
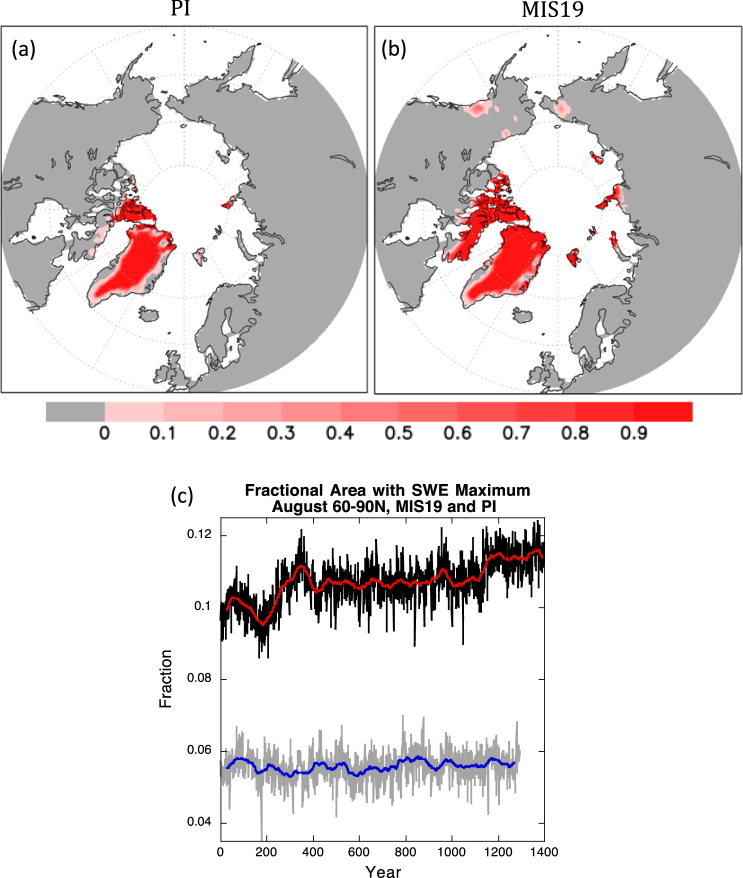
[Top]: Fraction of years during the final 50 years of the simulations when snow depth reaches the model-constrained 1 m SWE limit in (**a**) PI and (**b**) MIS19. [Bottom]: Time series from MIS19 (black) and PI (gray) simulations showing the fraction of surface area poleward of 60°N that reaches this limit during August. The 50-year running mean is shown in red for MIS19 and blue for PI.

Locations of incipient glaciation can also be identified as grid boxes that reach the model-constrained snow depth limit of 1 m snow water equivalent (see Methods). The areal extent of such regions doubles in MIS19 versus PI (including Greenland) and encompasses most of Baffin Island and Novaya Zemlya and all of Svalbard, Franz Josef Land, Severnaya Zemlya, and the New Siberian Islands (Fig. 4a,b). Many of these locations are regarded as initial nucleation sites for marine ice sheets that developed over the Barents and Kara Seas^22^. Moreover, the snow depth limit is reached inland on Taimyr Peninsula and in a few isolated regions in far northeastern Siberia and northwestern North America. Over the entire Arctic (60°–90°N), the area reaching maximum snow depth increases during the MIS19 simulation (Fig. 4c), suggesting an expansion of glaciation with time. By contrast, the corresponding area in PI is fairly stable and far smaller than in MIS19, evident by the clear separation in their time series.

Particularly interesting for this study is the emergence of maximum snow depth in MIS19 over most of the Canadian Archipelago and Baffin Island within the first few years of the simulation. This region has long been supposed to be the main area of glacial inception for the Laurentide ice sheet^23^, and there is clear evidence that glaciers expanded on Baffin Island during the Little Ice Age^24,25^. Our PI simulation corresponding to the end of the Little Ice Age shows most of the island with permanent snow cover, based on the 0.05 concentration, and occasional years when the maximum snow depth threshold is reached (Figs 3,4). This area and the expanded region of maximum snow depth along the Greenland coast in the MIS19 run are likely sources for ice-rafted debris deposited in MIS 19^3^. Although CCSM4 does not represent glacial processes, the model’s pronounced cooling and sea ice expansion in the North Atlantic and Labrador Sea would have created a favorably chilled marine environment for the production and preservation of calving icebergs, whose deposits associated with MIS19 glacial inception have been identified in sediments at North Atlantic Ocean Drilling Program Site 983^4^ south of Iceland, around the region of extreme temperature decreases (Fig. 2b).

Large areas of permanent snow cover blanket northeastern Eurasia, but no regions reach the snow depth limit east of the Taimyr Peninsula except near the Chuhotka Peninsula in easternmost Siberia. Glaciated landscapes in northeastern Russia are known to be limited to mountainous regions because of pervasive aridity and strong continentality with relatively warm summers^26^. There is evidence of a possible MIS5d local glacier advance in the Chuhotka Peninsula^27^ and Verkhoyansk Mountains of Siberia^28^ but not in other parts of northeast Siberia.

Although a colder climate in MIS19 is the proximate cause of permanent snow cover emerging in these three regions, the CCSM4 simulation reveals important contributions from circulation changes that appear to play a major role in shaping these patterns. Both increased accumulation of snowfall and reduced ablation act to enhance snow depth, but most studies have found that a decreased ablation effect is more important for generating permanent snow cover^29–31^.

**Figure 5:**
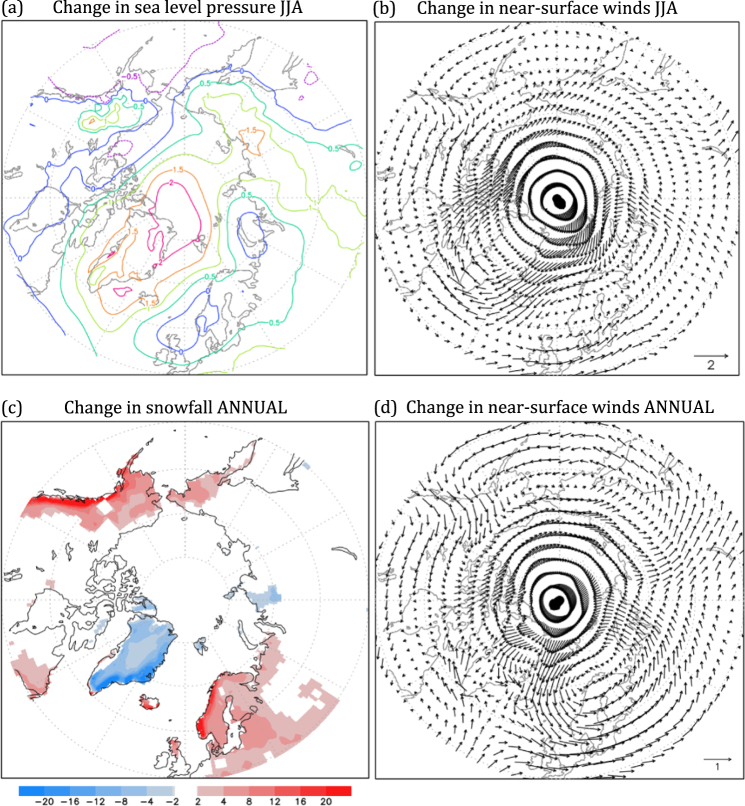
Changes between MIS19 and PI simulations. (**a**) Sea level pressure (hPa) and (**b**) lower atmosphere (surface-750 hPa) wind velocity (m s^−1^) in summer; (**c**) snowfall (cm) and (**d**) lower atmosphere (surface-750 hPa) wind velocity (m s^−1^) annually.

For glacial inception in MIS19, both processes contribute but their relative importance differs by region, as is the case for ice sheets generally^32^. Over Baffin Island and the Archipelago, reduced ablation is promoted by the development of a summertime high-pressure anomaly centered over the broader Greenland area that extends across much of the Arctic Ocean and into Siberia (Fig. 5a). This anomalous anticyclone is fostered by the strong surface cooling of the ocean surrounding Greenland (Fig. 2b), which in turn is caused by the combination of a weakened AMOC and expanded sea ice (Fig. 2c). This ice expansion results in a pronounced spread of sub-freezing surface conditions during summer in the North Atlantic-Labrador Sea region, approximately delineated by where sea ice cover expands by at least 15%. A consequence of the anomalous high pressure around Greenland is a change in the lower-atmospheric wind field (Fig. 5b). This results in enhanced flow over the much-colder ocean surrounding Greenland and onto Baffin Island, where the most pronounced emergence of maximum snow thickness occurs (Fig. 4). The circulation change over the Arctic Ocean also favors less ablation over Siberia, due to the weaker but consistently onshore flow coming from the Arctic Ocean.

Over northwestern North America, by contrast, the emergence of a perennial snowpack is fostered by atmospheric circulation changes that favor increased accumulation of snowfall through enhanced transport of onshore, upslope flow into the Rocky Mountains and Alaska (Fig. 5c,d). The large increase of annual snowfall over this region matches well with the appearance of permanent snow cover (Fig. 3). A similar type of circulation change also favors the increase of snowfall over the mountains of Norway, where a few grid cells become permanently snow covered in MIS19. However, the accumulation effect is not responsible for the development of permanent snow cover over Baffin Island, which receives slightly less snowfall in MIS19.

In summary, both our model simulations and proxy evidence suggest currently suitable conditions for incipient glaciation, in the absence of anthropogenic carbon emissions, due to the same favorable orbital and greenhouse forcing that triggered the cessation of interglacial warmth at the end of MIS19. Present-day orbital forcing is virtually the same as in MIS19, and contemporary GHG forcing would be virtually equivalent to MIS19’s if the Holocene climate had followed the expected late-interglacial GHG decline^33^. In that case, our present-day natural climate should be approximately the same as MIS19’s, including glacial inception. If the upward GHG trends during the late Holocene were caused by early agricultural carbon emissions, then ancient farming was apparently sufficient to avert a contemporary glacial inception.

**Figure 6:**
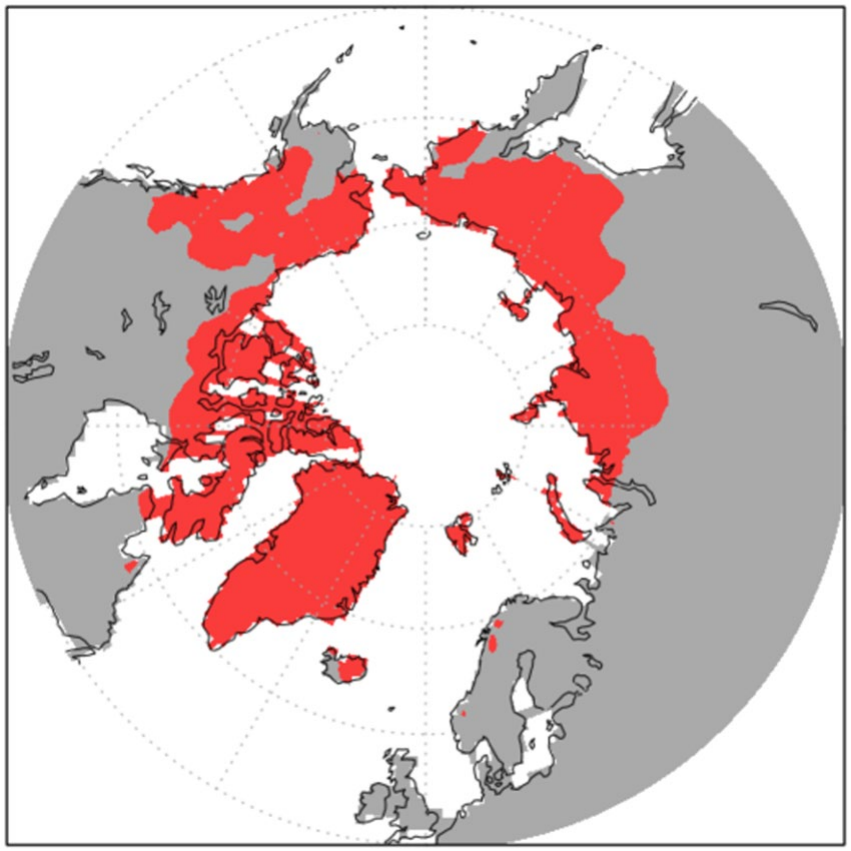
Regions with a year-round snow pack in PI_NAT, based on August snow cover of at least 5% in a gridbox.

Support for this possibility comes from our supplemental “Natural PI” CCSM4 model experiment, PI_NAT, which used contemporary orbital parameters and estimated natural GHG concentrations (Table 1; see Methods). Its expanded permanent snow cover (Fig. 6) is virtually identical to the MIS19 run (Table 2, Fig. 3b), indicating that the similarities in greenhouse forcing between MIS19 and “natural” present-day dominate over the slight orbital forcing differences.

**Table 2:** Comparison of Arctic (60–90°N) insolation forcing annually (W m^−2^), GHG radiative forcing (W m^−2^), permanent snow cover area (×10^6^ km^2^), and Arctic (60–90°N) temperature change (K) relative to PI among CCSM4 simulations.

Simulation	Insolation Forcing	GHG Forcing	Permanent Snow Cover	Arctic Temperature Change (K)
PI	—	—	5.99	—
PI_NAT	—	−1.02	9.93	−4.87
MIS19	−0.75	−0.92	9.92	−4.39
MIS5e	−4.3	0	8.57	−0.70

The simulation of MIS 5e (115ka) is from ref.^36^.

Although the PI_NAT results are based on estimated levels of greenhouse gases in the absence of all anthropogenic carbon emissions, these estimated concentrations can be partially constrained. Removing the CO_2_ contribution from industrialization lowers the contemporary concentration to 285 ppm, which therefore serves as an absolute upper limit for a natural present-day. This CO_2_ value was preceded, however, by rising concentrations since 8000 years ago, when the concentration had fallen to 260 ppm. Because CO_2_ levels in all recent, comparable interglaciations identified by ref.^34^. (MIS 5, 7, 9, 11, 17, and 19) declined during their final eight millennia (up to their insolation minimum), an expected present-day CO_2_ concentration is no higher than 260 ppm. In fact, CO_2_ values have fallen by a minimum of 5 ppm (MIS 7 and MIS 11) and a maximum of 16 ppm (MIS 9 and MIS 17) in their final 8000 years. This spread implies an expected contemporary concentration of CO_2_ as low as 244 ppm and sets another plausible upper limit of 255 ppm, bracketing a range that encompasses the 245 ppm concentration used in PI_NAT as our best estimate of a natural present-day value (see Methods).

That a fairly small CO_2_ decrease, relative to the CO_2_ rise from industrialization, can leverage a large climate change is supported by findings of enhanced cold-climate sensitivity. Stronger positive feedbacks between temperature and albedo (in this case illustrated by a pronounced expansion of sea ice area and permanent snow cover) occur in relatively cold climates, such as those with greenhouse gas concentrations only slightly below those of PI^35^, implying that only a modest external forcing perturbation is then required to push the climate system into glacial inception. Further evidence for this enhanced cold climate sensitivity is found in comparisons of external forcing changes and climatic responses in the four CCSM4 simulations summarized in Table 2. PI_NAT has only modestly reduced GHG forcing relative to PI (−1.02 W/m^2^), but there is a large increase in permanent snow cover and a large decrease in Arctic surface temperature^35^. MIS 19 has only a small net negative Arctic insolation forcing (−0.75 W/m^2^) and a slightly smaller reduction in GHG forcing relative to PI (−0.92 W/m^2^), yet the response is an equally large increase in permanent snow cover and nearly as large a decrease in Arctic temperature as in PI_NAT (Table 2).

In contrast, the glacial inception simulation of ref.^36^ for 115 ka (the end of MIS 5e) employs a much larger annual negative insolation forcing (−4.3 W/m^2^) in the Arctic (60–90°N) with no change in GHG forcing relative to PI and produces a large increase in permanent snow cover (although not quite as large as PI_NAT or MIS 19), along with a small decrease in Arctic annual temperature. The large orbitally forced decrease in summer temperature is sufficient to initiate the increase in permanent snow cover, even with GHG forcing equivalent to PI (the annual Arctic temperature decrease is smaller because the winter temperatures are higher). These experiments thus demonstrate “bookends” of forcing required for glacial inception: either strong orbital forcing with no change in greenhouse gases (MIS5e) or strong greenhouse forcing with little or no orbital influence (MIS19, PI_NAT) relative to present-day. Glacial inception occurs in all three cases, but cold climate sensitivity via a strengthened positive temperature/albedo feedback is most enhanced for PI_NAT and MIS 19, whose GHG forcing is slightly reduced relative to PI.

## Discussion

There are a number of caveats relevant to our conclusions. First, because there is no accepted definition of glacial inception, we developed our own to identify a major expansion of permanent snow cover. By requiring complete separation in the time series of hemispheric areal coverage between the equilibrated PI and MIS19 climates, we believe that this condition constitutes a fairly strict criterion to define incipient glaciation. However, we also recognize that any such measure is subjective and thus vulnerable to alternative definitions.

Second, simulations of snow cover are highly dependent on variations in model topography, which cannot be fully represented in global climate models of ~1° horizontal resolution used here. This limitation is especially relevant in regions with cold climates and high elevations and thus becomes particularly important for MIS19. The expansion of permanent snow cover in a cooling climate can be strongly muted in coarse-resolution models that are unable to resolve small-scale topographic variations in mountainous areas^37,38^. Therefore, the inferred expansion of glaciation in our MIS19 simulation may be a conservative estimate.

Third, the GHG concentrations used in the MIS19 experiment are subject to dating errors and interpolation uncertainties in selecting the most accurate values corresponding to a somewhat subjective date of 777 ka for the close of MIS19. However, we have used the recently updated^6^ estimates for CO_2_, and our target date agrees well with the end of MIS19 inferred from a recent high-precision ^40^Ar/^39^Ar chronology^4^. Differences in estimated GHG concentrations within a millennium of our target 777 ka date are small (2–4 ppm CO_2_ and 10–50 ppb CH_4_) and result in minor radiative forcing differences within 0.10 W m^−2^.

Fourth, CCSM4 does not include ice sheet dynamics and relies on a crude way of accounting for glacial calving that constrains the buildup of a snowpack. This limitation necessitates the use of less precise indicators of glacier formation, such as where snow cover persists above a threshold and where it reaches the maximum allowable depth in the model. These indicators are thus simplifications of complex glacier processes, although our chosen sub-gridscale fractional threshold results in a 20^th^ century CCSM4 simulation that agrees with observations of residual August snow cover^39,40^, and we expect that small-scale topographic variations promote patchy snow cover in excess of the broader regional average.

Fifth, CCSM4 has a cold bias in northern Canada and northern Siberia^41^ that predisposes the model toward forming permanent snow cover in a cooler climate. However, these are mostly cold-season biases^36^; in spring-summer, the terrestrial cold bias is smaller, while the Canadian Archipelago even has a weak warm bias^42^. Nevertheless, the model produces too much snow cover in its 20^th^ century transient simulation over Alaska, the Rocky Mountains, and much of northern Canada (including Baffin), which are glacial inception regions in the model^42^.

A confluence of recent studies has greatly improved our understanding of glacial inception during MIS19, with respect to chronology, greenhouse forcing, and climate conditions, although there exist very few detailed observational records for evaluating our MIS19 model simulation. This study bolsters the body of evidence on the late MIS19 climate, when orbital conditions were very similar to present day. Our GCM simulations also significantly advance previous modeling^5^ by allowing a higher-resolution fingerprint of glacial inception using a more complete representation of climatic processes. The high resolution reveals important dynamical responses involving circulation changes combined with topographic influences that promote glacial inception through the emergence of permanent snow cover regionally. The model suggests that altered circulation in northeastern Canada and Siberia fosters nucleation via reduced ablation, while anomalous onshore and upslope flow from the Pacific Ocean favors glaciation in the Rocky Mountains via enhanced accumulation. Similar responses were found in a previous study with lowered GHG and shown to be directly attributable to higher model resolution^37^, whereas the simplified geography and coarse resolution of EMICs are unable to capture these regional changes. To the extent that these dynamical alterations stem from characteristic surface cooling patterns in colder climates, our findings provide new physical insights into the processes responsible for glacial inception generally. For example, prior research^29^ suggested that altered atmospheric circulation was critical for the inception of the Laurentide Ice Sheet by 116 ka, but that study was unable to identify the relevant physical mechanisms.

This study builds on our own prior research through a novel investigation of the EAH by simulating an *actual* climate that should have strongly resembled a contemporary climate devoid of anthropogenic carbon emissions, rather than only relying on estimated boundary conditions, as in PI_NAT. Follow-up investigations will benefit from ongoing model developments to explicitly simulate ice sheet dynamics^43^ and increase resolution to even more realistically account for small-scale topographic variations and associated microclimates important for glacial nucleation. Furthermore, there is a pressing need to expand the coverage and spatiotemporal resolution of the very limited observational data for MIS19. In parallel, these kinds of advances in modeling and reconstructions will shed new light on the processes responsible for the end of the MIS19 interglacial climate and the vulnerability of contemporary climate to glacial inception in the absence of anthropogenic interference.

## Methods

### Model description

To estimate the climate at the end of MIS19, we employ the widely used Community Climate System Model Version 4 (CCSM4), a fully coupled global climate model^41^. The horizontal resolution in the land and atmospheric components is 1.25° latitude × 0.9° longitude, and the atmosphere is resolved into 26 layers. The ocean and sea ice models use variable grid spacing from 0.27° to 0.54° meridionally and uniform 1.11° zonally, while employing 60 vertical levels in the ocean. CCSM4 has one of the most accurate representations of the contemporary climate system^44^, although it does have a cold, snowy bias in high northern latitudes^36^. This model has been used in paleoclimate studies of the mid-Holocene^45^, Last Millennium^46^, Glacial Inception^36^, and Last Glacial Maximum^47,48^. We have used CCSM4 and its predecessor versions in prior modeling studies of the EAH^37,39,49,50^.

### Simulated snow cover and sea ice

Of particular importance in our study of cold-climate processes in MIS19 is the representation of snow cover and sea ice. CCSM4’s land model^51^ simulates snow cover extent reasonably well but is biased toward early melt^52^ and tends to underestimate snow depth. The simulated snowpack is treated as a one-dimensional vertical column that accounts for accumulation and melting of fallen snow, as well as compaction and transfer of water between snow layers^53^. Fractional snow coverage within a grid box depends on snow density and snow depth, thus allowing for realistic patchiness of snow cover. To prevent unbounded snow buildup in the absence of an ice sheet dynamics model, CCSM4 limits the accumulation of snow depth to 1 m snow water equivalent. Any snowfall that exceeds this limit is converted into its equivalent fresh water volume and transferred to the ocean to ensure salinity balance.

The sea ice component^54,55^, includes improvements in depicting radiative transfer, melt ponds, and ice dynamics. CCSM4 is among the best-performing models in simulating Arctic sea ice, due in part to its sophisticated treatment of melt ponds, a sub-gridscale ice thickness distribution, and improved solar radiation physics^55^.

### Model scenarios

Our MIS19 simulation was integrated for 1420 years, initialized from a long control run using fixed boundary conditions for the pre-industrial climate at year 1850^41^. Approximate equilibrium occurred by year 1150, when the top-of-atmosphere radiative balance was achieved and global mean surface temperatures stabilized. We compared the final five decades of the MIS19 simulation to a reference climate state of the last 50 years of a 1300 year-long pre-industrial run, “PI”, driven with modern orbital parameters and GHG concentrations matching those that occurred in year 1850 (Table 1). Because of the lower GHG concentrations at 777 ka compared with 1850, which we attribute to the absence of early agricultural emissions in MIS 19, the greenhouse radiative forcing is nearly 1 W m^−2^ lower in the MIS19 simulation.

To remove the estimated influence of pre-industrial carbon emissions from early farming, we ran an additional experiment, PI_NAT, which represents our best estimate of the contemporary climate system in the absence of both industrialization and ancient agriculture. The orbital forcing of these two year-1850 experiments is identical, but PI_NAT uses GHG concentrations based on estimates of pre-industrial carbon emissions from archeological and paleoecological data (see below) and levels determined in our previous studies^37,39^ (Table 1). The resulting greenhouse radiative forcing is only slightly more negative (−0.12 W m^−2^) in PI_NAT than in the MIS19 experiment.

An ancillary simulation of the present-day climate was also used for comparison (Table 1). This transient “20^th^ century” run was begun from the equilibrated, pre-industrial model climate of 1850 and integrated to 2005 using observed boundary conditions of solar irradiance, volcanoes, aerosols, and greenhouse gas concentrations^41^. The final 20 years of this experiment is taken to represent contemporary climatic conditions that include anthropogenic warming contributions from both industrialization and agriculture.

### Greenhouse gas concentration estimates

The concentrations of CO_2_ and CH_4_ derived for a natural Holocene are based on agreement between “top-down” and “bottom-up” evidence^34^. The top-down estimate is derived from extrapolated GHG concentrations from their early-mid Holocene values and is consistent with the average fall in CO_2_ and CH_4_ during previous interglaciations. The interglacial average concentration of CO_2_ and its envelope of interglacial spread provide strong evidence that the late Holocene CO_2_ rise far exceeded expected bounds as early as 2,000 years ago (see Supplementary Information). The bottom-up estimate is obtained from carbon emission budgets from archeological and paleobotanical data. For example, the spread of irrigated rice in southeast Asia since the middle Holocene probably contributed most of the CH_4_ increase from the mid-Holocene by 1000 years ago, with additional emissions caused by the spread of livestock and greater biomass burning of weeds and crop residues^34,56^. Likewise, the estimated 40 ppm reduction in CO_2_ between PI and a natural contemporary value is based on revised land-use reconstructions^57^ combined with new insights on historical per-capita land clearance^58^. The resulting estimates of pre-industrial, anthropogenic carbon emissions account for a 24 ppm CO_2_ difference, which swells to approximately 40 ppm when augmented by additional carbon outgassing from a warmer ocean^34^.

### Glacial inception

For purposes of this study, we identify glacial inception in MIS19 if the simulated Northern Hemisphere snow cover area remaining at the end of the summer melt season (August) in all 50 years exceeds the corresponding hemispheric snow cover area in all 50 years of PI. In keeping with our previous studies^59^, we consider a grid cell to be snow covered if its areally averaged snow depth is at least 0.01 m. Based on the model’s parameterized conversion of grid-cell averaged snow depth to fractional coverage^60^, this depth limit translates to at least 5% snow cover. Furthermore, we find that this definition yields a simulated year-round snow cover area in the late 20^th^ and early 21^st^ centuries that nearly matches observations^40^. We also apply a more stringent alternative condition for defining incipient glaciation: regions where the simulated snow accumulation reaches the model-constrained maximum of 1 m snow water equivalent. Such locations imply glacial growth that is thwarted by the limits imposed by CCSM4. Utilizing both definitions provides a more complete perspective of glacial nucleation sites.

### Data availability

All model output is archived on the National Center for Atmospheric Research’s High Performance Storage System (HPSS) and is publicly available via the NCAR Data Sharing Service (https://www2.cisl.ucar.edu/resources/storage-and-file-systems/using-the-ncar-data-sharing-service).

## Electronic supplementary material

Supplementary Information
