# Supplementary material for: Glacial Inception in Marine Isotope Stage 19: An Orbital Analog for a Natural Holocene Climate
Source: Sci Rep. 2018 Jul 5;8:10213. doi: 10.1038/s41598-018-28419-5 (PMC6033942; doi:10.1038/s41598-018-28419-5)
Supplement: Supplementary file 1 — Supplementary Information [file 41598_2018_28419_MOESM1_ESM.pdf]

Glacial Inception in Marine Isotope Stage 19: An Orbital Analog for a Natural Holocene Climate

S. J. Vavrus, F. He, J. E. Kutzbach, W. F. Ruddiman, and P. C. Tzedakis

Comparison of possible Holocene analogs

Because the low eccentricity and precession (εsinω) values in MIS11 were a good match to those in the Holocene, MIS11 has been used to suggest that the CO_2 increase and the lack of glacial inception during the late Holocene is due to weak eccentricity and the associated weak reduction of summer insolation[1,2]. However, examination of the evidence from MIS19 argued against this conclusion[3], because there was no prolonged interglacial during MIS19 even though its low eccentricity was similar to that of MIS11[4]. Instead, another critical factor in glacial inception is the relative timing of the precession and obliquity signals: the precession and obliquity cycles were in phase during MIS19 but out-of-phase during MIS11 (Figure S1). In this respect, MIS19 is considered the best analog of the Holocene (MIS1) during the past 800,000 years for which ice-core records are available[4,5].

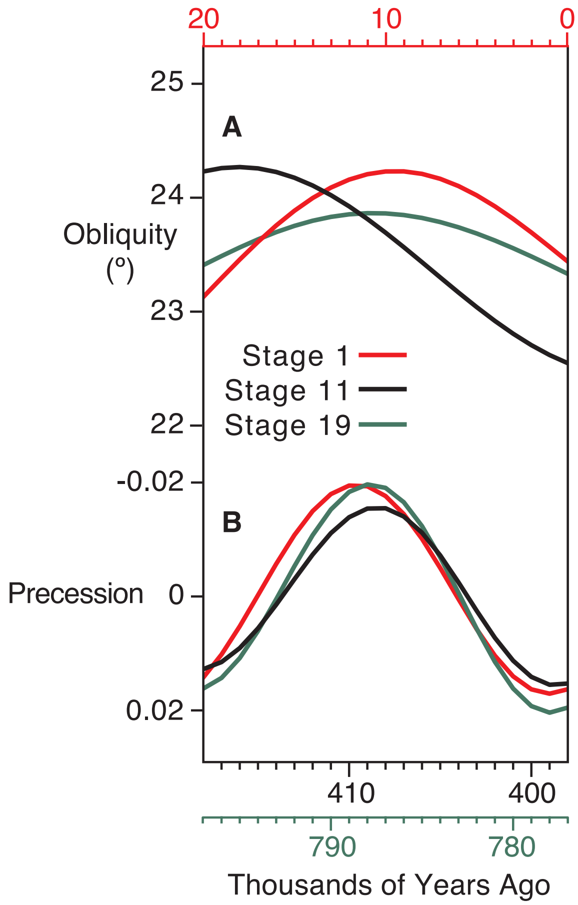

Supplementary Figure S1. Comparison of (a) obliquity and (b) precession (εsinω) during MIS19 (green), MIS11 (black), and MIS1 (red). Note: maximum obliquity and negative precession values[6] equate to maxima in Northern Hemisphere summer insolation.

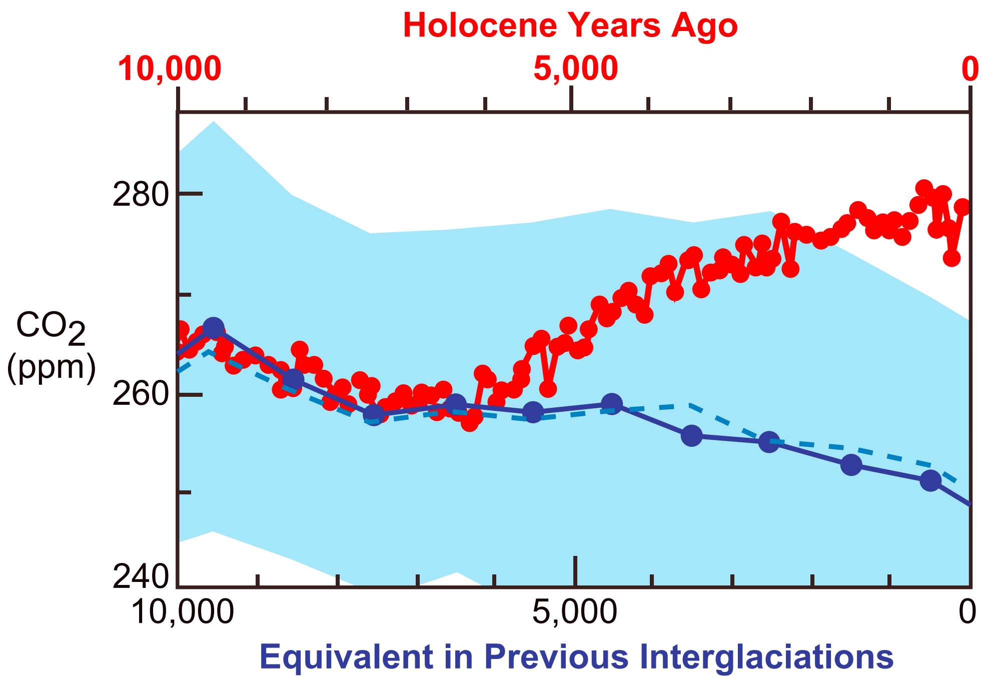

Supplementary Figure S2. Trend in CO_2 during the Holocene (red) versus the final 10,000 years prior to insolation minimum among previous interglaciations, expressed as the interglacial average (blue lines) and one standard deviation (light blue shading). MIS15 is included in the average shown by the dashed line and excluded in the trend depicted by the solid line. Data sources are the same as in Figure 1. [From Ref.[7]]

References

1 EPICA community members*. Eight glacial cycles from an Antarctic ice core. *Nature* **429**, 623-628, doi:10.1038/nature02599 (2004).

2 Broecker, W. S. & Stocker, T. F. The Holocene CO_2 rise: Anthropogenic or natural? *Eos, Transactions American Geophysical Union* **87**, 27-27, doi:10.1029/2006eo030002 (2006).

3 Jouzel, J. *et al.* Orbital and millennial Antarctic climate variability over the past 800,000 years. *Science* **317**, 793-796, doi:10.1126/science.1141038 (2007).

4 Tzedakis, P. C., Channell, J. E. T., Hodell, D. A., Kleiven, H. F. & Skinner, L. C. Determining the natural length of the current interglacial. *Nature Geoscience* **5**, 138-141, doi:Doi 10.1038/Ngeo1358 (2012).

5 Yin, Q. Z. & Berger, A. Individual contribution of insolation and CO2 to the interglacial climates of the past 800,000 years. *Climate Dynamics* **38**, 709-724, doi:10.1007/S00382-011-1013-5 (2012).
6 Past Interglacials Working Group of Pages. Interglacials of the last 800,000 years. *Reviews of Geophysics* **54**, 162-219, doi:10.1002/2015RG000482 (2016).
7 Ruddiman, W. F. *et al.* Late Holocene climate: Natural or anthropogenic? *Reviews of Geophysics* **54**, 93-118, doi:10.1002/2015RG000503 (2016).
